# Prescribing Trends for Acne Vulgaris Visits in the United States

**DOI:** 10.3390/antibiotics12020269

**Published:** 2023-01-28

**Authors:** Patrick O. Perche, Gabrielle M. Peck, Lillian Robinson, Ayman Grada, Alan B. Fleischer, Steven R. Feldman

**Affiliations:** 1 Center for Dermatology Research, Department of Dermatology, Wake Forest School of Medicine, Winston-Salem, NC 27104, USA; 2 College of Medicine, University of Cincinnati, 3230 Eden Ave., Cincinnati, OH 45267, USA; 3 Department of Dermatology, Case Western Reserve University School of Medicine, Cleveland, OH 44106, USA; 4 Department of Dermatology, University of Cincinnati, 3230 Eden Ave., Cincinnati, OH 45267, USA; 5 Department of Pathology, Wake Forest School of Medicine, Winston-Salem, NC 27101, USA; 6 Department of Social Sciences & Health Policy, Wake Forest School of Medicine, Winston-Salem, NC 27101, USA

**Keywords:** acne vulgaris, prescription, over the counter, dermatology, family medicine, pediatrics, NAMCS, National Ambulatory Medical Care Survey, database

## Abstract

Acne vulgaris is the most common reason for pediatric patients and third most common reason for adult patients to seek care from a dermatologist in the US. However, referring providers may be reluctant to initiate patients on acne treatment or certain prescriptions. We assessed over-the-counter (OTC) and prescription acne (antibiotic and non-antibiotic) treatment rates to characterize differences by patient demographics and provider specialty. The National Ambulatory Medical Care Survey (NAMCS) was analyzed for all acne therapies prescribed for at least 10 unweighted visits between 1993 and 2016 (most recent years available). Prescription rates varied by age, with younger patients more likely to receive a prescription; insurance status, with privately insured patients more likely to receive a prescription; and across and within specialties, with dermatologists more likely to recommend a prescription medication than family medicine and pediatric providers. Among all forms of antibiotics for acne vulgaris, oral minocycline was the most commonly prescribed antibiotic by dermatologists, followed by oral doxycycline. Oral minocycline was also the most common antibiotic prescribed by family physicians, followed by oral doxycycline and oral clindamycin, respectively. Pediatricians appeared to be less likely to prescribe oral antibiotics for acne. The OTC topical antimicrobial benzoyl peroxide was the most utilized drug for acne among pediatricians, and it was also the most commonly recommended OTC drug for acne among dermatologists, family physicians, and pediatricians.

## 1. Introduction

Acne vulgaris is the most common reason for pediatric patients and third most common reason for adult patients to seek care from a dermatologist in the US [1]. However, referring providers may be reluctant to initiate patients on acne treatment or certain prescriptions [2]. Many patients utilize over-the-counter (OTC) acne treatments prior to or in lieu of visiting a medical provider, and now more than ever, there are a multitude of efficacious OTC treatment options, mainly for mild-to-moderate acne [3,4]. These include the retinoid adapalene, benzoyl peroxide (BPO), and alpha and beta hydroxy acids, among others [4]. Not infrequently, multiple products are necessary to achieve satisfactory results, and regimens often consist of both OTC and prescription acne medications [5].

The early initiation of effective acne treatment is crucial to prevent permanent physical scarring and psychosocial sequelae [6]. Acne can cause patients significant psychological and social impairment, regardless of disease severity [6]. Specifically, higher levels of isolation, depression, and anxiety, and lower levels of self-worth and body satisfaction are associated with acne [6]. When persisting into adulthood, those with acne have higher unemployment rates than those without acne [6]. Moreover, adults with acne appear to suffer from higher rates of anxiety and depression when compared to adolescents [7]. This is thought to be partly due to sociocultural beliefs that consider acne a disease of adolescence [7]. In light of this, willingness to prescribe appropriately aggressive acne treatment and a patient-centered approach is warranted to prevent long-term sequelae.

The pathophysiology of acne involves an interplay of androgen-induced sebum overproduction, altered keratinization, inflammation, and bacterial colonization of the pilosebaceous unit by *Cutibacterium acnes* (*C. acnes*) [5]. *C. acnes* induces inflammation via the activation of toll-like receptors in macrophages and keratinocytes, and reductions in colony counts are directly correlated with clinical improvement [4,5]. Thus, antimicrobials targeting the colonization of *C. acnes* has been implicated in many treatment algorithms [8,9,10]. Considering the abundance of therapeutics available to patients and providers in treating this disease, we assessed the variability in acne therapies prescribed by provider specialty and patient demographics. Specifically, we assessed OTC and prescription acne (antibiotic and non-antibiotic) treatment rates to characterize differences by patient demographics and provider specialty in the fields of dermatology, family medicine, and pediatrics.

## 2. Results

There were 765,400 records for acne vulgaris, estimating the experience of 21.1 billion acne visits over the study period (Supplementary Table S2). Isotretinoin was only included in the leading five therapies for dermatologists and was not included in the five most common therapies amongst pediatricians and family medicine providers (Table 1). Benzoyl peroxide was the most commonly recommended OTC treatment overall.

Upon comparing prescription and OTC acne treatment rates, family medicine providers and dermatologists were more likely to recommend a prescription acne treatment than an OTC acne treatment (Odds Ratio (OR): 4.59 [95% Confidence Interval]: [3.53–5.92]; *p* < 0.0001 and OR: 7.18 [6.13–8.41]; *p* < 0.0001, respectively). The prescribing rates for OTC and prescription acne treatments did not differ for pediatricians (Table 2). Dermatologists were more likely than family medicine providers and pediatricians to recommend a prescription medication (OR: 10.9 [8.4–14.2] and 3.8 [3.1–4.7]; *p* < 0.0001 for both); family medicine providers were more likely than pediatricians to recommend a prescription acne medication (OR: 2.9 [2.1–3.9]; *p* < 0.0001).

Patients < 18 years old were more likely to receive a prescription medication than those ≥ 18 years old (OR: 1.44 [1.23–1.68]; *p* < 0.0001). Moreover, patients with private insurance were more likely to receive a prescription than those with Medicare (OR: 1.51 [1.08–2.11]; *p* < 0.05), while there was no difference for patients with private insurance versus Medicaid and Medicaid versus Medicare.

There were no statistically significant prescription rate differences when stratified by sex, race, or geographic region, except for the greater odds of receiving a prescription if living in the Southern vs. Western US (OR: 1.24 [1.01–1.53]; *p* < 0.05; Table 2).

## 3. Materials and Methods

The National Ambulatory Medical Care Survey (NAMCS) is a survey which provides objective, reliable information about non-hospital-based ambulatory medical services in the United States (US). The Division of Health Care Statistics at the National Center for Health Statistics (NCHS) of the Centers for Disease Control and Prevention (CDC) conducts the survey annually and receives response rates of up to 77%. The NAMCS has been used previously by our research group in order to characterize trends in dermatology [11,12,13].

We conducted a population-based, cross-sectional analysis using the NAMCS between 1993 and 2016, the most recent years available. We examined all acne therapies that were prescribed by dermatologists, family practitioners, and pediatricians at ≥ 10 unweighted visits with a listed diagnosis of acne, per specialty, during the study period. To do so, first we stratified acne visits by specialties of interest (dermatology, family practitioners, and pediatricians). Within those stratified groups, we then excluded acne therapies that were prescribed at less than 10 unweighted visits. These therapies were excluded as it would be difficult to ascertain accurate results from a sample size of less than 10 unweighted visits. Because the NAMCS survey allows for up to 30 medications and 5 different diagnoses to be listed per visit, not all therapies in the original dataset were exclusively acne therapies. Thus, a subsequent list of medications prescribed at visits where acne was the only listed diagnosis was pulled from the database, and any medications not on the acne-only therapy list were excluded from the original list of therapies. Acne therapies were subsequently assigned as either prescription or OTC based on expert opinion of the authors and status of the acne therapy at the time of analysis (Supplementary Table S1). Survey procedures in SAS v9.4 were used to compare the odds ratio of a prescription or OTC product being prescribed when stratified by provider specialty, patient age, patient gender, region of the United States, and insurance status (SAS Institute Inc., Cary, NC, USA). Significance was set at *p* < 0.05, and 95% Confidence Intervals were generated. A two-tail test was used to determine significance. The five most common medications prescribed for acne were also analyzed for dermatology, family medicine, and pediatrics, using the weighted frequencies from NAMCS for those therapies that met the inclusion criteria. The prevalence was calculated by taking the weighted frequency for a therapy and dividing by the sum of the weighted frequencies as stratified for each specialty.

During a randomly selected week of the year, physicians and advanced practice providers (nurse practitioners and physician assistants) are selected at random by the NCHS to participate in the NAMCS survey. Participation in the survey is confidential and voluntary. Specifically, the sample is selected by utilizing a three-stage process. First, 112 geographic areas (counties, townships, or equivalents) in the US are sampled, and then physicians and advanced practice providers are selected from master files from the American Medical Association (AMA) and the American Osteopathic Association (AOA). Finally, for each healthcare provider, a proportion of visits from a one-week period from the year is randomly selected for the sample. Visit sampling rates vary from 20% to 100% based on the patient volume of the practice. For each visit that is included in the sample, physicians and advanced practitioners document demographic information, diagnoses, medications prescribed, and procedures performed for each visit. The NAMCS captures up to 30 medications and 5 diagnoses for each visit sampled. Weighting factors accounting for time and geographic variability are assigned in order to derive nationally representative estimates [14]. A multistage estimation procedure is employed to produce unbiased national estimates and includes four components: (i) inflation by reciprocals of the probabilities of selection, (ii) nonresponse adjustment, (iii) a ratio adjustment to fixed totals, and (iv) weight smoothing [15].

The NCHS’s Ethics Review Board (ERB) is the only Institutional Review Board (IRB) which must review the NAMCS. The ERB has attained IRB approval for the NAMCS, and thus we did not need to seek approval from our institution for this study.

## 4. Discussion

Prescription rates varied by age, with younger patients more likely to receive a prescription; insurance status, with patients having private insurance more likely to receive a prescription; and across and within specialties, with dermatologists more likely to recommend a prescription medication than both family medicine and pediatric providers.

OTC and prescription acne medications have respective advantages and disadvantages, serving important roles in the management of acne. In a study of 529 participants assessing the general public’s experience with acne treatment, their preferences, and barriers to care, 43% of respondents reported barriers to accessing medical care for their acne [3]. Cost and transportation were the top two most common reasons. Of respondents that have used both OTC and prescription acne treatments, 58% preferred prescription treatments compared with 29% preferring OTC treatments (13% no preference) [3]. Lower cost, ease of accessibility, and fewer side effects were reasons in favor of OTC treatments. Those favoring prescription treatments reported greater efficacy and oversight by a medical professional as advantages [3].

Among antibiotics for acne vulgaris, oral minocycline was dermatologists’ most commonly prescribed antibiotic, followed by oral doxycycline. Oral minocycline was also the most common antibiotic prescribed by family physicians, followed by oral doxycycline and oral clindamycin, sequentially. Pediatricians appeared to be less inclined to prescribe oral antibiotics for acne. The OTC topical antimicrobial BPO was the most commonly utilized antibiotic for acne among pediatricians. This, in part, may be due to fewer side effects associated with topical antimicrobials compared with oral antibiotics, particularly in children.

BPO is an organic peroxide, an oxidant, with keratolytic, comedolytic, and bactericidal properties that has been used to treat acne since the 1930s and was the most commonly recommended OTC treatment in our study [4]. It is available OTC in concentrations of 2.5–10%; however, 2.5% is as efficacious as the 5% and 10% concentrations for mild-to-moderate acne with less cutaneous side effects, such as desquamation, erythema, and burning (another potential adverse effect is bleaching clothing or other fabrics exposed to the chemical) [4]. By acting as a potent oxidizing agent that creates reactive oxygen species, BPO reduces levels of *C. acnes* and coagulase-negative *Staphylococcus aureus*, and it is efficacious against resistant strains of *C. acnes* [4,16]. The global prevalence of antibiotic-resistant *C. acnes* rose from 20% in 1978 to 60% in 1996 [4]. Resistance is most frequently associated with oral erythromycin, tetracycline, and clindamycin. However, doxycycline, trimethoprim, and minocycline resistance have increased as usage has increased [4,17]. BPO is frequently combined with topical clindamycin due to the synergistic effect in reducing both resistant and non-resistant *C. acnes* strains [4]. In the last decade, BPO has also been combined with topical retinoids, which have shown to be efficacious, raising the point of whether topical antibiotics are necessary [16]. Nonetheless, topical antibiotic monotherapy is not recommended per the American Academy of Dermatology acne guidelines due to the risk of bacterial resistance [18]. BPO has no known risk of creating resistant *C. acnes*, in contrast to topical and oral antibiotics [16].

The Global Alliance to Improve Outcomes in Acne raised attention to the need for antibiotic stewardship in its 2009 acne guidelines [9]. Recommendations included limiting the use of oral antibiotics to 3 months, using topical retinoids for maintenance, and pursuing aggressive treatment when necessary to limit acne scarring [9]. The recommendations were effective in reducing the quantity of antibiotics prescribed by dermatologists, with a reduction of 36.6% over the years 2008–2016 [19]. From 2003 to 2013, the length of antibiotic courses also decreased, with a median of 345 days in 2003 to 126 days in 2013 [20]. While antibiotic usage decreased in that time period, the use of hormonal acne treatments such as the antiandrogen spironolactone is on the rise and saw a 391% increase in prescribing rates [20]. There has been continued emphasis on antibiotic stewardship in the 2016 American Academy of Dermatology acne guidelines and the 2018 Global Alliance acne guidelines [10,18]. With increased focus on antibiotic stewardship and shorter courses of oral antibiotics, it is likely that the trends in acne prescribing will continue to evolve over the next several years.

Retinoids are a staple in the dermatologist toolbox for the treatment of acne. In our study, topical retinoids were the top two drugs prescribed by dermatologists and made the top three for both family practitioners and pediatricians. Topical retinoids are considered first-line therapy for acne and have comedolytic, anti-inflammatory, antiproliferative, and immunomodulatory effects [21]. First-generation retinoids, such as tretinoin and isotretinoin (oral), are FDA-approved to treat acne and, while frequently used, are also limited by cutaneous and systemic toxicity, respectively [21]. Second-generation retinoids include acitretin (oral), which is FDA-approved for the treatment of severe psoriasis in adults [21]. Third-generation retinoids, such as adapalene and tazarotene, are topical medications that are also used to treat acne [21]. Adapalene was FDA-approved to treat acne in 1996 and is available in 1% and 3% strengths [21]. The 1% strength is available OTC. Adapalene is considered to have less cutaneous side effects than tretinoin [21]. Not infrequently, patients discontinue topical retinoids due to skin irritation, prompting escalation in therapy, such as oral antibiotics [22]. While adapalene may be slightly less efficacious than tretinoin, the diminished risk of irritation is likely to increase patient satisfaction and medication adherence, reducing the need for antibiotics [22,23]. Additionally, in some trials, the two medications are considered similarly effective [24]. In retinoid-naïve patients, starting with adapalene over tretinoin is reasonable.

While less frequently recommended by providers in our study, products containing salicylic acid are widely available OTC and efficacious primarily for non-inflammatory, comedolytic acne or mild inflammatory acne. Salicylic acid is a lipophilic β-hydroxy acid with moderate comedolytic and desmolytic properties, promoting corneocyte desquamation [4]. Salicylic acid also decreases sebum production, contributing to its anti-acne therapeutic effects [25]. These properties are concentration-dependent, and OTC salicylic acid is commonly available in 0.5% to 5% concentrations. Higher concentrations (20–30%^+^) are used for chemical peels and are only recommended for use under the supervision of a medical professional [25]. The most common side effects include cutaneous irritation and mild peeling; however, it is usually minimal [4].

Limitations of our study include that the NAMCS database does not provide an exact quantity for the frequency of various acne therapeutics prescribed by providers; rather, the data are based on survey responses from outpatient visits over one week of the year and extrapolated to a much larger scale. The data included in our study from the NAMCS database were over a 23-year period from 1993 to 2016 and may not reflect current clinical behavior. The most recent years were not included due to a publication lag of the data in the NAMCS database. However, acne therapies were classified as prescription or OTC based on the therapy status at the time of our data analysis to reflect current trends best. A limitation of therapy classification is that a few therapies are available as both prescription and OTC. Of all the acne therapies included in our study, to our knowledge, only one therapy, adapalene, changed in classification (prescription only to OTC and prescription) during the study period (in 2016). Acne severity was not controlled in our analysis, but it may influence prescribing trends, as dermatologists frequently see and treat the severe spectrum of disease, whereas primary care physicians may see milder or earlier presentations of disease.

Awareness of prescribing rates may allow for the optimization of care. Dermatologists may be more comfortable with chronically managing certain prescription acne treatments, such as oral isotretinoin [26,27]. Currently, a vast majority of providers prescribing isotretinoin are dermatologists [28]. Emphasis on evidence-based guidelines for acne treatment and on primary care providers in initiating isotretinoin may reduce healthcare costs and improve disease outcomes through increased access to care for patients with severe or refractory acne [27,29]. Nonetheless, a patient-centered approach with patients actively involved in the therapeutic decision is necessary to achieve therapeutic success.

## Figures and Tables

**Table 1:** Top 5 medications prescribed for acne vulgaris in the US according to the National Ambulatory Medical Care Survey, 1993–2016, by dermatology, family medicine, and pediatrics.

Top 5 Medications Prescribed for Acne in the US, from 1993 to 2016, Using NAMCS											
Dermatology				Family Medicine				Pediatrics			
Treatment	Route	N (Millions)	Prevalence (Percent)	Treatment	Route	N (Millions)	Prevalence (Percent)	Treatment	Route	N (Millions)	Prevalence (Percent)
Tretinoin	Topical	7.52	11.5	Minocycline	Oral	1.52	26.9	Benzoyl Peroxide	Topical (OTC)	0.947	20.7
Isotretinoin	Oral	7.24	11.1	Tretinoin	Topical	1.21	21.4	Minocycline	Oral	0.844	18.4
Minocycline	Oral	5.80	8.86	Doxycycline	Oral	0.699	12.4	Tretinoin	Topical	0.783	17.1
Benzoyl Peroxide	Topical (OTC)	5.47	8.36	Clindamycin	Oral	0.697	12.4	Doxycycline	Oral	0.585	12.8
Doxycycline	Oral	5.00	7.65	Benzoyl Peroxide	Topical (OTC)	0.481	8.53	Benzoyl Peroxide-Clindamycin	Topical	0.558	12.2

**Table 2:** Odds ratios comparing prescription and over-the-counter (OTC) rates by specialty and patient demographic for acne vulgaris visits in the National Ambulatory Medical Care Survey, 1993–2016.

Odds Ratios Comparing Prescription and Over-the-Counter Rates for Acne	
Outcome of Interest	Odds Ratio (OR)
Specialty	
Family Medicine Rx vs. OTC	4.59 [3.53–5.92]; *p* < 0.0001
Dermatology Rx vs. OTC	7.18 [6.13–8.41]; *p* < 0.0001
Pediatrics OTC vs. Rx	1.08 [0.88–1.33]; *p* = 0.5
Dermatology vs. Family Medicine for Rx	10.9 [8.4–14.2]; *p* < 0.0001
Dermatology vs. Pediatrics for Rx	3.8 [3.1–4.7]; *p* < 0.0001
Family Medicine vs. Pediatrics for Rx	2.9 [2.1–3.9]; *p* < 0.0001
Patient Age	
<18 years old vs. ≥18 years old for Rx	1.44 [1.23–1.68]; *p* < 0.0001
Patient Insurance Type	
Private Insurance vs. Medicare for Rx	1.51 [1.08–2.11]; *p* < 0.05
Private Insurance vs. Medicaid for Rx	1.37 [1.00–1.88]; *p* = 0.05
Medicaid vs. Medicare for Rx	1.08 [0.75–1.56]; *p* = 0.7
Patient Sex	
Male vs. Female for Rx	0.95 [0.81–1.11]; *p* = 0.5
Patient Race	
White vs. Black for Rx	1.29 [0.89–1.9]; *p* = 0.2
Patient Ethnicity	
Hispanic vs. Non-Hispanic for Rx	1.04 [0.61–1.79]; *p* = 0.9
Region	
Midwest vs. West US for Rx	1.23 [0.99–1.52]; *p* = 0.07
Northeast vs. West US for Rx	1.02 [0.80–1.30]; *p* = 0.9
South vs. West US for Rx	1.24 [1.01–1.53]; *p* < 0.05
South vs. Midwest US for Rx	1.02 [0.84–1.23]; *p* = 0.9
South vs. Northeast US for Rx	1.22 [0.98–1.52]; *p* = 0.08
Midwest vs. Northeast US for Rx	1.29 [0.96–1.51]; *p* = 0.1

## Data Availability

Publicly available datasets were analyzed in this study. This data can be found here: https://ftp.cdc.gov/pub/Health_Statistics/NCHS/Datasets/NAMCS/, accessed on 1 December 2022.

## Supplementary Materials

The following supporting information can be downloaded at https://www.mdpi.com/article/10.3390/antibiotics12020269/s1. Supplementary Table S1: List of medications utilized for acne vulgaris visits with at least 10 unweighted visits between 1993 and 2016 in the National Ambulatory Care Survey (NAMCS) database. Medications were categorized as either prescription (Rx) or over the counter (OTC) for coding with SAS statistical software v9.4. Supplementary Table S2: Patient demographic data. Based on 765,400 records estimating 21.1 billion visits for acne vulgaris in the National Ambulatory Medical Care Survey from 1993 to 2016.
