# Supplementary material for: Prescribing Trends for Acne Vulgaris Visits in the United States
Source: Antibiotics (Basel). 2023 Jan 28;12(2):269. doi: 10.3390/antibiotics12020269 (PMC9952425; doi:10.3390/antibiotics12020269)
Supplement: Supplementary file 1 [file antibiotics-12-00269-s001.zip › antibiotics-2122593-supplementary.pdf]

antibiotics

Supplementary File

Table S1. List of medications utilized for acne vulgaris visits with at least 10 unweighted visits between 1993-2016 in the National Ambulatory Care Survey (NAMCS) database. Medications were categorized as either prescription (Rx) or over-the counter (OTC) for coding with SAS statistical software v9.4.

Medications	Treatment Type
adapalene-benzoyl peroxide topical	Rx
amoxicillin	Rx
ampicillin	Rx
azelaic acid topical	Rx
benzoyl peroxide-clindamycin topical	Rx
cephalexin	Rx
clindamycin	Rx
clindamycin topical	Rx
clindamycin-tretinoin topical	Rx
clobetasol topical	Rx
dapsone topical	Rx
desoximetasone topical	Rx
doxycycline	Rx
drospirenone-ethinyl estradiol	Rx
erythromycin	Rx
isotretinoin	Rx
ketoconazole	Rx
minocycline	Rx
nitrogen	Rx
spironolactone	Rx
sulfacetamide sodium ophthalmic	Rx
sulfacetamide sodium-sulfur topical	Rx
sulfamethoxazole-trimethoprim	Rx
tazarotene topical	Rx
tetracycline	Rx
tretinoin topical	Rx
triamcinolone	Rx
triamcinolone topical	Rx
adapalene topical	OTC
benzoyl peroxide topical	OTC
ceteareth; dimethicone; glycerin topical; hyaluronic acid	OTC
hydroquinone topical	OTC
ointment hydrophilic	OTC
petrolatum topical	OTC
salicylic acid topical	OTC
topical acne agents	OTC

Table S2. Patient demographic data. Based on 765,400 records estimating 21.1 billion visits for acne vulgaris in the National Ambulatory Medical Care Survey from 1993-2016.

Demographics	Percent of Responses
Sex	
Male	42.1%
Female	57.9%
Age	
<18	31.8%
≥18	68.2%
Race	
Caucasian, Non-Hispanic	71.0%
African-American, Non-Hispanic	10.7%
Hispanic	13.2%
Other Race/Multiple Race	5.1%
Insurance	
Private	62.2%
Medicaid	16.0%
Medicare	11.6%
Other	10.2%
Region	
Northeast	19.4%
Midwest	23.5%
South	32.2%
West	24.9%
